# Clinical implications of the universal definition for the prevention and treatment of heart failure

**DOI:** 10.1002/clc.23842

**Published:** 2022-07-05

**Authors:** Chanchal Chandramouli, Simon Stewart, Wael Almahmeed, Carolyn Su Ping Lam

**Affiliations:** ^1^ National Heart Centre Singapore Singapore Singapore; ^2^ Duke‐National University of Singapore Singapore Singapore; ^3^ Torrens University Australia Adelaide South Australia Australia; ^4^ University of Glasgow Glasgow UK; ^5^ Institute of Health Research University of Notre Dame Australia Fremantle New South Wales Australia; ^6^ Institute of Cardiac Science, Sheikh Khalifa Medical City Abu Dhabi United Arab Emirates; ^7^ Heart and Vascular Institute, Cleveland Clinic Abu Dhabi United Arab Emirates; ^8^ University Medical Centre Groningen Groningen The Netherlands

**Keywords:** clinical syndrome, HFpEF, HFrEF, NT‐proBNP, stakeholders, symptoms and signs, universal definition of heart failure

## Abstract

The diagnosis of heart failure (HF) primarily relies on signs and symptoms that are neither sensitive nor specific. This impedes timely diagnosis and delays effective therapies or interventions, despite the availability of several evidence‐based treatments for HF. Through monumental collaborative efforts from representatives of HF societies worldwide, the universal definition of HF was published in 2021, to provide the necessary standardized framework required for clinical management, clinical trials, and research. This review elaborates the key concepts of the new universal definition of HF, highlighting the key merits and potential avenues, which can be nuanced further in future iterations. We also discuss the key implications of the universal definition document from the perspectives of various stakeholders within the healthcare framework, including patients, care providers, system/payers and policymakers.

## 1 INTRODUCTION

Heart failure (HF) is a clinical syndrome with a myriad of etiologies and underlying pathophysiology. With more than 64 million people afflicted worldwide, the prevalence of HF is increasing rapidly within the aging populations of high‐income countries.^1^ The total direct medical cost of HF accounted for an estimated US$31 billion in 2012 and is projected to increase by ~127% to $69.7 billion by 2030.^2^ Evidence‐based interventions exist yet progress in achieving target doses of guideline‐directed medical therapies (GDMTs) or disrupting the cascade toward worsening HF has not been effective.^3^, ^4^ The substantive burden of morbidity and mortality imposed by HF continues to worsen worldwide, with key economic disparities limiting the capacity of healthcare systems to respond.^5^ Logically, quantifying the burden of disease of any condition and then comparing the capacity of different communities to achieve better outcomes is predicated on a universal definition of the condition in question.

Unfortunately, there is global ambiguity and subjectivity in the interpretation and definition of what constitutes HF. Such variability has impeded the implementation of therapeutics in clinical practice, communication with patients to facilitate a shared decision‐making approach, and further understanding of the pathology from a research standpoint. In response, leaders from the Heart Failure Society of America, Heart Failure Association of the European Society of Cardiology, Japanese Heart Failure Society, Heart Failure Association of India, Cardiac Society of Australia and New Zealand, and the Chinese Heart Failure Association took the monumental step of proposing a universal definition of HF in 2021.^6^ This review introduces the key concepts and potential advantages surrounding this definition whilst highlighting opportunities for further refinement. In doing so, we consider the stakeholders who will be instrumental in the successful implementation of this new universal definition of HF.

### 1.1 Heart failure definitions—An evolving concept

**Figure 1:**
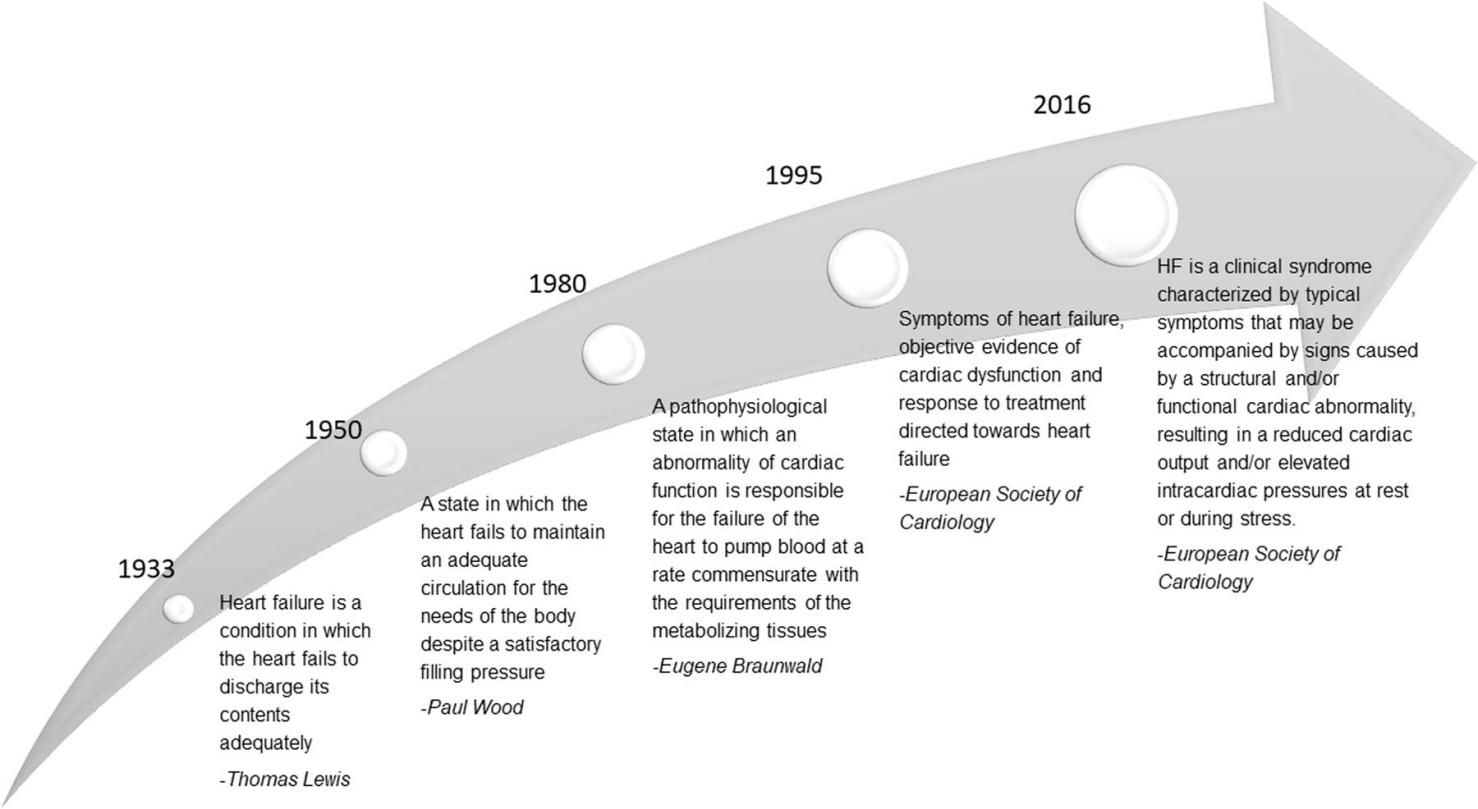
Evolution of heart failure definitions.

As highlighted in Figure 1, the definition of HF has significantly evolved and expanded over time. Early definitions referred to it as “a condition in which the heart fails to discharge its contents adequately,”—essentially confining the case definition to those presenting as HF with reduced ejection fraction (HFrEF).^7^ Today, HF is typically, but not uniformly, defined as a clinical syndrome (i.e., recognizable patterns of signs and symptoms) that is related to any functional and/or structural cardiac impairment, resulting in reduced cardiac output and/or elevated intracardiac pressure at rest or during stress.^8^, ^9^, ^10^, ^11^, ^12^ Yet, for research purposes, case definitions of HF (e.g., Framingham criteria) include an objective left ventricular ejection fraction (LVEF) threshold, New York Heart Association (NYHA) functional class, previous hospitalization, and sometimes even natriuretic peptides are preferred for clinical trial enrolment.^6^

### 1.2 Shortcomings of current HF definitions

Current definitions of HF fall short in several ways. First, the hemodynamic component of the definition (i.e., reduced cardiac output or filling pressure) is subjective, making it difficult to implement in the public healthcare setting. Despite elevated N‐terminal prohormone of brain natriuretic peptide (NT‐proBNP) levels receiving the highest class recommendations to support the diagnosis of HF^9^, ^12^ and featuring as a common inclusion criterion in recent large‐scale clinical trials, it is visibly absent from HF definitions.^6^ This contrasts with the definition of myocardial infarction (MI), whereby the presence of elevated plasma levels of a key biomarker (high‐sensitivity troponin) is an instrumental feature within the universal definition of MI and pivotal to establishing a clinical diagnosis that triggers definitive treatment.^13^ Conversely, the clinical diagnosis of HF chiefly relies on vague signs (pulmonary crackles, peripheral edema) and symptoms (dyspnea on exertion, fatigue) that are not always objective, sensitive, or specific.^9^ Not only does it make it difficult for clinicians to establish a definitive diagnosis and rapidly initiate effective treatment strategies, the nonspecific signs are also confusing for patients who may not recognize signs of onset/worsening of HF and only present to critical care units at a more advanced stage.^14^ Such confusion extends to non‐HF specialists, such as primary care physicians who receive little guidance specific to their scope of practice to optimize the early detection and management of the syndrome.^15^ Lack of standardized definition in diagnosis in clinical trials and endpoints collection such as HF‐related death and hospitalizations also limits the scientific merits and clinical research potential.^16^ Therefore, a new, simple, unified definition for HF is not just a nicety, but a necessity to calibrate clinical management, entry criteria in clinical trials, and case definitions for population research.

### 1.3 New universal definition

HF is defined as a clinical syndrome with symptoms and/or signs caused by structural and/or functional cardiac abnormality and corroborated by elevated natriuretic peptides and/or objective evidence of pulmonary or systemic congestion.^6^

**Figure 2:**
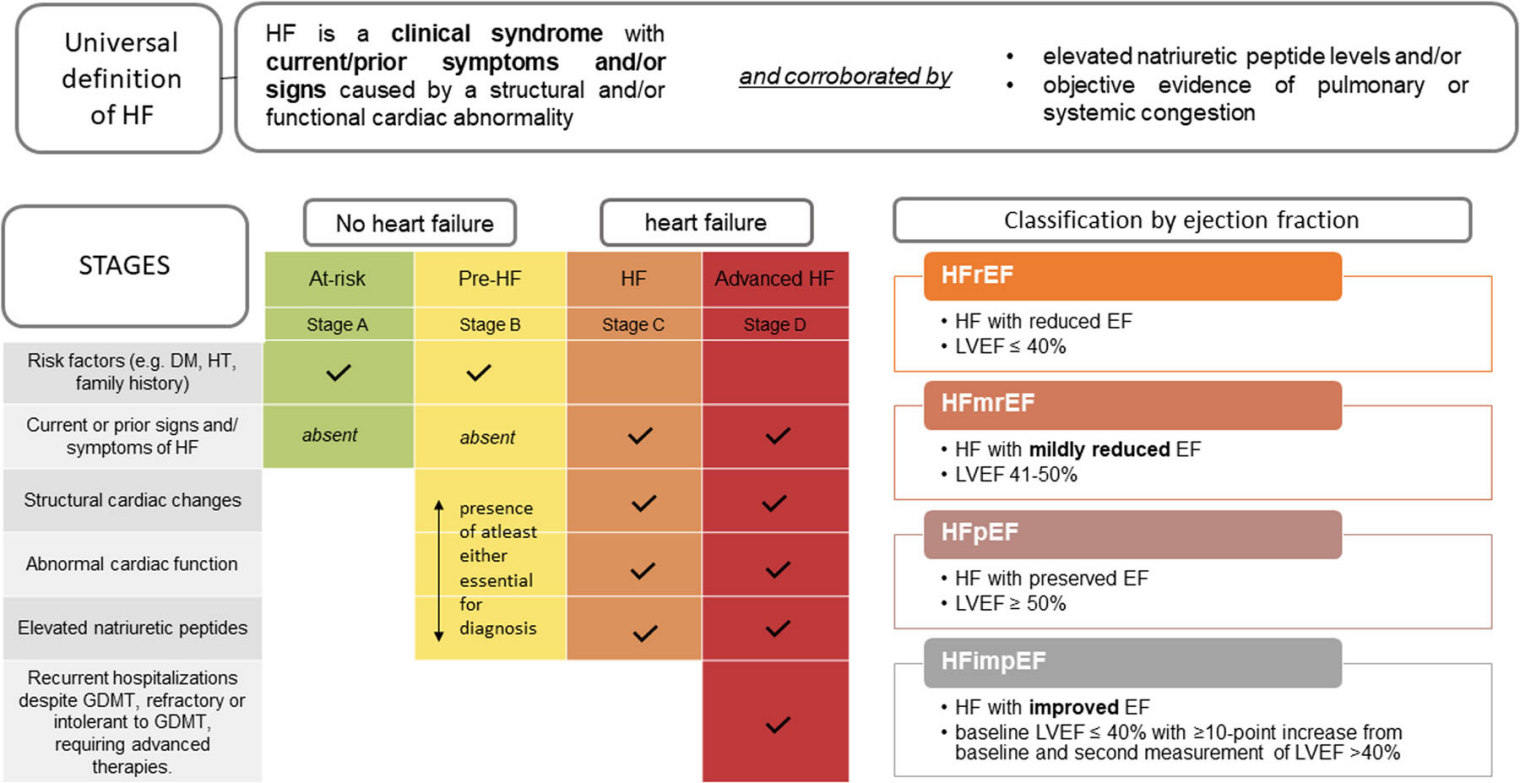
Key concepts of a universal definition of HF. DM, diabetes mellitus; EF, ejection fraction; GDMT, guideline‐directed medical therapy; HF, heart failure; HT, hypertension; LVEF, left ventricular ejection fraction.

As described above, a new universal definition of HF (see text box) was recently proposed as part of the consensus document. The universal definition of HF, introduced in 2021, elegantly described HF as above. A new HF staging approach was also proposed Namely, at risk for HF, pre‐HF, HF, and advanced HF in place of the previous stages A–D articulated by the American Heart Association. “At risk for HF” refers to individuals at risk for HF, but without current or prior symptoms or signs of HF and without structural or biomarkers evidence of heart disease; “pre‐HF,” individuals present without current or prior symptoms or signs of HF, but have evidence of structural heart disease or abnormal cardiac function, or elevated natriuretic peptide levels; whilst “HF” patients are defined as those with current or prior symptoms and/or signs of HF caused by a structural and/or functional cardiac abnormality; finally, “advanced HF” patients present with severe symptoms and/or signs of HF at rest, recurrent hospitalizations despite GDMTs, and refractory or intolerant to GDMTs, requiring advanced therapies. Importantly, presymptomatic individuals (i.e., at risk for HF and pre‐HF) are no longer grouped together with those considered to have developed the syndrome of HF (Figure 2).

The universal definition also proposes a categorical grouping of LVEF based on treatment differences (Figure 2) as follows:

I. HFrEF, LVEF ≤ 40%;
II. HF with mildly reduced ejection fraction (HFmrEF), LVEF of 41%–to 49%; “mildly reduced” replacing “mid‐range”;
III. HFpEF, LVEF ≥ 50%; and
IV. HF with improved ejection fraction (HFimpEF): baseline LVEF ≤ 40% with ≥10‐pt increase from baseline and second LVEF > 40%.

One of the key changes includes categorizing LVEF of 40% and below as HFrEF (previous classification: LVEF < 40% as HFrEF), in recognition of trials evaluating important therapeutic targets that have included those with LVEF of 40% as HFrEF and showed therapeutic benefit (e.g., PARADIGM‐HF, EPHESUS‐HF, MERIT‐HF, DAPA‐HF, TRED‐HF). To some extent, this bodes well in recognizing patients with HFrEF who may otherwise be missed from the practice of rounding up LVEF to the nearest 5% in echocardiographic lab reports.^17^, ^18^ Although uncommon, subjects with measured LVEF of 35% may have 40% recorded, making their clinical report semiquantitative. In addition, HF with “mid‐range” EF has been replaced with HF with “mildly reduced” EF, while retaining the same acronym of HFmrEF. The heterogeneous etiological characteristics of patients with HFmrEF mean that some have profiles synonymous with HFpEF and others with HFrEF. However, the neurohormonal blockade has proven to be effective in patients with more reduced than preserved LVEF and has benefits in patients with HFmrEF in CHARM,^19^ PARADIGM‐HF & PARAGON‐HF,^18^ and BB‐meta‐HF.^20^ Potentially, with this renaming of HFmrEF, the historical exclusion of these patients from clinical trials will be rectified. Most importantly, for the first time, changes in LVEF trajectory have been formally recognized in the revised staging. Improvement in the LVEF can be observed with optimal GDMT, particularly in patients with HFrEF; in contrast, progressive decline in LVEF could indicate of poor prognosis warranting intensification of therapies.^21^ Accordingly, whilst single time‐point evaluation of LVEF provides important prognostic information on a sex‐specific basis,^22^ it neither captures nor informs the clinical trajectory of patients with HF, disallowing effective HF management.

## 2 MERITS AND STRENGTHS

The universal definition serves as a necessary consensus definition of HF to standardize the recognition of HF across clinical care and clinical trials. Key merits associated with the universal definition and the new staging are elaborated further in this section.

### 2.1 Utilization of biomarkers for an objective diagnosis of HF

Biomarker‐based diagnosis is becoming increasingly common for disease diagnostic and definition purposes, including the universal diagnosis of MI.^13^ Estimated glomerulation filtration rate in chronic kidney disease and hemoglobin A1c in diabetes have, respectively, served similar purposes. In the context of the ongoing conundrum around how best to prevent the syndrome,^23^ in the 2017 ACC/AHA/HFSA focused update, biomarker NT‐proBNP‐based screening in those at risk of developing HF received a Class IIa recommendation (level of evidence: B–R). Those with elevated NT‐proBNP levels are strongly recommended to be managed by team‐based care including a cardiovascular specialist and GDMT to prevent the development of LV dysfunction (systolic or diastolic) or new‐onset HF.^11^ Yet, the diagnosis of HF to date primarily relies on vague, nonspecific signs and symptoms (e.g., fatigue, breathlessness, and ankle swelling, etc.).^8^, ^9^, ^12^ Consequently, patients who dismiss these nonspecific symptoms only present at emergency care or to specialists at a much‐advanced stage of HF, leaving little or no room for early interventions. Natriuretic peptides are early and robust markers of congestion, even in the absence of symptoms of HF. The inclusion of natriuretic peptides as part of the new definition establishes an objective diagnostic standard for HF because they are (1) myocardium‐specific, (2) dependent on intracardiac volumes and filling pressures, (3) elevated before symptoms/signs of HF surface, and (4) relevant to a range of cardiac abnormalities, including and not limited to the systolic function, diastolic function, valvular heart disease, right HF, and atrial fibrillation.^24^

Contention remains over the utility of NT‐proBNP as an HF diagnosis “rule‐in” marker because other clinical conditions, besides HF, also affect NT‐proBNP levels. Increased NT‐proBNP levels are also observed in chronic kidney disease, atrial fibrillation, pericardial disease, pulmonary embolism, and, broadly, within the context of the aging heart.^6^ While elevated NT‐proBNP is an excellent marker of congestion, further investigations on clinical history, blood tests, and imaging will be required to identify cardiac dysfunction for a conclusive diagnosis. Alternatively, obesity, treatment with diuretics and constrictive pericarditis (less common) are associated with reduced NT‐proBNP levels.^4^ Indeed, obesity is common in HF and the inverse relationship between BMI and NT‐proBNP is evident in both the context of HFrEF and HFpEF.^25^, ^26^ However, low NT‐proBNP levels neither rule out the diagnosis^27^ nor predict treatment response in both HFrEF^28^ and HFpEF.^29^ Individual data from 12 763 patients with HFrEF in the BIOS (Biomarkers In Heart Failure Outpatient Study) consortium show that NT‐proBNP independently predicts 5‐year all‐cause mortality, even with a paradoxical reduction in absolute NT‐proBNP levels with increasing BMI categories (3785, 2193, 1554, 1045, 755, and 879 ng/L, for underweight, normal weight, overweight, and mildly, moderately, and severely obese patients, respectively).^30^ Overall, although not perfect, NT‐proBNP still retains excellent diagnostic potential and independent prognostic value in HF. Thus, it is particularly useful in nearly every clinical context, including primary care.

### 2.2 Noncardiovascular entities that mimic HF

There are various noncardiovascular entities that mimic HF, as standalone or coexisting pathology with HF. Renal failure, liver failure, anemia, severe obesity with peripheral edema, and chronic respiratory failure hypoventilation syndrome are some of these competing diagnoses of HF. In acute care settings, patients presenting with congestion and acute dyspnea are often misclassified as HF when often confounded or caused by anemia or iron deficiency.^6^, ^31^ Similarly, congestion could be primarily due to chronic kidney disease with little evidence of cardiac dysfunction, requiring very different treatment and management practices.^32^ The new universal definition not only recognizes these entities but also emphasizes the importance of distinguishing HF from mimicking conditions, by including discriminating elements that are specifically HF‐related.

### 2.3 Demarcation of presymptomatic patients without HF from those with HF

The consensus document formally recognizes those at risk for HF and pre‐HF as “no HF” to avoid the stigma of HF before symptom onsets. Critically, this encourages targeted HF prevention strategies. Asymptomatic stages of HF with patients at risk of HF (formerly referred to as Stage A) and patients with cardiac structural disease or abnormal cardiac function or elevated NPs (former Stage B) are no longer categorized as having HF. Interestingly, the relabeling of HF staging shares several common elements with the TNM staging system (0–IV) for cancer.^33^ The TNM‐like HF staging proposed by the Universal Definition Committee is simple, clinically useful, and efficient when used to plan a therapeutic strategy (especially prevention).

Approximately 1.1 million individuals are hospitalized for HF annually, while 6.2 million are diagnosed with HF and 573 million are at risk of HF or pre‐HF.^34^ The prevalence estimates from a random sampling of adults (*n* = 2029) from Olmsted County, Minnesota, showed that 32% were healthy without HF, 22% in Stage A, 34% in Stage B, 12% in Stage C, and 0.2% in Stage D HF.^35^ The magnitude of the population at risk for progression to overt HF is huge (46%),^35^ but aggressive risk factors control efforts among these individuals are missing. Data from National Health and Nutrition Examination Surveys (NHANES) between 2007 and 2010 (≥20 years, *N* = 4470) demonstrated that Stage A HF is poorly recognized and inadequately managed despite one in three American adults (mean age 56.9 years; 51.5% women) having Stage A HF.^35^ In addition, the current International Classification of Diseases (ICD) codes to identify HF (ICD‐10‐CMP 142.9 and ICD‐10‐HF 150) only capture Stage B, C, and D. The lack of ICD codes for patients at risk for HF or pre‐HF has neither allowed systematic monitoring nor reporting of these conditions so far.^36^ Standardized definitions and revision of the current HF continuum nomenclature in the universal definition document help delineate individuals who are “at risk,” “asymptomatic,” and “symptomatic HF” to comprehensively characterize and treat the specific risks.

### 2.4 Recognition of LVEF and clinical trajectory

One of the most important aspects of the new staging definition is the recognition of LVEF and clinical trajectory. LVEF is a dynamic measure of cardiac pump function, as is glomerular filtration rate to renal function. Longitudinal changes in LVEF hold important prognostic implications in HF.^21^, ^37^, ^38^ With optimal GDMT, patient adherence, and reverse remodeling, improvement in LVEF is common, especially among HFrEF. Similarly, added comorbidities and poor adherence to medications also may cause transitions from HFpEF to HFmrEF and HFrEF. Conventionally, patients with an increased LVEF are referred to as either “improved” LVEF or borderline HFpEF or HF with recovered EF (HFrecEF). However, single‐handed reliance on LVEF quantification for the optimal management of the syndrome may be confounded by intra‐ and inter‐reader variabilities. Migration from one LVEF category to another over time based on small changes in EF needs cautious interpretation as it can have important implications for HFmEF, particularly on a sex‐specific basis given that women may have a slightly worse prognosis than men with the same LVEF around this clinically important threshold.^22^

Clinical status‐based assessments also inform the risk of HF hospitalization or mortality. The Expert Consensus provides a detailed course of action toward optimal therapy based on clinical trajectory during admission for HF, that is, for improved versus stalled after initial response versus not improved/worsened clinical status.^39^ The universal definition proposes the usage of the terminology “remission” instead of “recovered” to avoid therapeutic complacency and inappropriate withdrawal of treatment.^6^ Evidence from the TRED‐HF study showed that patients with dilated cardiomyopathy who were presumed to have “recovered” (LVEF > 50%, normal LV end‐diastolic volume index, NT‐proBNP < 250 ng/L, NYHA Class I) did not actually fully recover. Instead, 36% relapsed on withdrawal of GDMT.^40^ The terminology “persistent” instead of “stable” HF is also recommended in patients who do not show improvement to account for their high residual risk for clinical deterioration and death. The lack of improvement should be viewed as “worsening HF” and further GDMT optimization should be initiated If symptoms continue to worsen despite the escalation of therapies, patients can be viewed as “refractory to treatment” and referred for mechanical circulatory support, cardiac transplant, or even palliative care.^6^

### 2.5 Emphasis on prevention and risk factor control

The universal definition now allows early detection of patients with high risk for HF as candidates for primary prevention. This is important given that recent longitudinal cohort studies with extensive clinical and cardiac function phenotyping have shed more light on which individuals are most at risk of developing the syndrome.^41^ Active risk factor interventions can now be actioned in patients identified to be at risk of HF (former Stage A) based on underlying conditions such as hypertension, diabetes, obesity, exposure to cardiotoxins, familial cardiomyopathy, and so on. Nonpharmacological management with behavioral counseling interventions to promote a healthy diet and physical activity are highly recommended to control blood glucose, blood pressure, cholesterol, body weight, and smoking.^8^, ^9^, ^42^ Accumulating evidence on Dietary Approaches to Stop Hypertension diet interventions for effective lowering of low‐density lipoprotein cholesterol and blood pressure has received endorsements from the USDA Dietary Guidelines for Americans and AHA/ACC.^43^ Despite several expert panels recommending a multifaceted approach, which includes diet, lifestyle modification and pharmacological therapy, evidence‐based nutrition strategies have not yet become the standard of care in HF due to a lack of evaluations in pragmatic clinical trials.^44^ Whether such trials will prove successful is uncertain given the mixed results of equivalent trials of multifaceted primary care^45^ and nurse‐led, multidisciplinary care^46^ strategies in HF prevention.

## 3 OPPORTUNITIES FOR REFINEMENT AND FUTURE RESEARCH

The consensus document has excellent clinical utility potential with diagnostic, prognostic, and therapeutic validity. As the writing committee plans to refine the universal definition periodically, this offers opportunities to refine the definition of HF further to best guide management strategies. Here, we highlight a few areas that we will be anticipating further guidance on. Particularly, with regard to a more detailed classification of HFpEF, appropriation of congestion values based on clinical characteristics, LVEF in different populations and evolving automated diagnostic capabilities.

### 3.1 Focus on etiology, but not cardiac abnormality for HFpEF

The universal definition of HF requires the presence of structural and/or functional abnormalities. Typically, the echocardiographic abnormalities that underpin an HFpEF diagnosis, whilst of potential prognostic significance,^47^ include ventricular hypertrophy, left atrial enlargement, and mitral annular velocities. However, these parameters are neither sensitive nor specific in the context of HFpEF, as evidenced by large‐scale trials. Nearly a third of patients enrolled in I‐PRESERVE and TOPCAT had normal diastolic function (31% and 34%, respectively).^48^, ^49^ Although atrial enlargement is viewed as the litmus test for diastolic dysfunction in HFpEF, only mild Grade 1 diastolic dysfunction (29% and 22%) and mild atrial enlargement (51% and 19%) were noted in both studies, respectively.^48^, ^49^ The limitations of echocardiographic evaluations of diastole also add to the subjectivity in structural/functional abnormalities in defining the syndrome of HFpEF.^50^ Whether strain‐based measures or stress tests provide a better estimate of diastolic function in HFpEF remains to be elucidated. Perhaps, a greater emphasis on understanding the underlying etiologies over echocardiographic abnormalities in HFpEF syndrome evaluation could be highlighted in further iterations of the consensus document. For example, a recent coding structure in patients with suspected HFpEF suggests accounting for noncardiovascular entities as primary causes of congestion (“HF mimics”) first, then searching for an underlying etiology (“HFpEF attributed to”), and in the absence of an identifiable etiology, the contributing comorbidities are recognized (“HFpEF associated with”).^31^

### 3.2 Estimated cardiac congestion scores

Contemporary guidelines already recommend using natriuretic peptides in initial screening to diagnose HF. However, they do not recommend using them as a primary confirmatory diagnostic tool.^8^, ^9^, ^12^ While NT‐proBNP is almost always elevated in HF, there are other cardiovascular disorders (e.g., acute coronary syndrome, myocarditis, arrhythmia) and noncardiovascular conditions (e.g., aging, renal disease, sepsis, anemia, liver disease) that affect BNP/NT‐proBNP levels as well. NT‐proBNP levels are also lower in the presence of obesity (increased BMI) and in pericardial disease. A standardized cut‐off of NT‐proBNP may not be adequate across different age, sex, and ethnic groups. An individual‐participant data meta‐analysis of 95 617 subjects without a history of CVD in 40 prospective studies showed that plasma concentration of NT‐proBNP is higher in women (vs. men) and increases, indicative of declining diastolic ventricular and renal function.^51^ In fact, a higher NT‐proBNP threshold has been proposed in older patients and in women.^52^ This requires further validation in clinical trials and practice. Whether a higher threshold in older people is clinically meaningful or merely limits healthcare access specifically needs to be further elucidated. Sex‐specific threshold adjustments may be of clinical value, although the underlying pathobiology of women having higher plasma BNP/NT‐proBNP is not understood. Differences in body composition, fat distribution, hormonal influence, and menopausal status have been suggested as potential explanations.^53^ Emerging evidence also suggests racial and ethnic differences in circulating NT‐proBNP levels, with lower levels observed among Blacks, Hispanics, and Asians than Whites in healthy and asymptomatic HF cohorts.^54^, ^55^, ^56^ Therefore, interpretations of NT‐proBNP values should not stand alone but also account for key clinical covariates. Consequently, research efforts should be geared toward the creation of a detailed NT‐proBNP‐based congestion algorithm with adjustments for key clinical correlates, including age, sex, ethnicity, obesity, renal function, heart rhythm, and so on.^52^

### 3.3 Population‐specific LVEF cut‐offs

Several studies have reported on the bimodal distribution of LVEF among patients with incident HF. Contemporary American and European echocardiography guidelines recognize that the normal LVEF range is shifted to (54%–74% in women vs. 52%–74% in men).^57^, ^58^ Recent evidence from pooled analyses of 13 195 patients with HF enrolled in the PARADIGM‐HF and PARAGON‐HF across the LVEF spectrum showed that the therapeutic effects of sacubitril/valsartan varied by EF and sex (*p*_interaction_ = 0.02). As supported by a large cohort study of sex‐specific thresholds of mortality,^22^ the beneficial effects of the neurohormonal blockade could potentially extend to patients with HFmrEF and possibly to an even higher range of LVEF in women.^16^ The EchoNoRMAL study has also derived age‐, sex‐ and ethnic‐appropriate adult reference values in population studies.^18^ Therefore, sex‐ and ethnicity‐specific LVEF cut‐offs in future revisions of the consensus document will provide the necessary biological premise, which is currently lacking.

### 3.4 Variability in LVEF measurement

The clinical and epidemiological utility of LVEF is well‐established but the accuracy of LVEF measurement in clinical practice is debatable. Echocardiography, the most widely used imaging modality of LVEF, incurs an 18%–21% intraobserver and 6%–13% interobserver variability.^59^ The inherent digit rounding bias, visual underestimation of true LVEF values, poor image quality, and measurement error collectively contribute to the misclassification of patients.^60^ In the TOPCAT trial, core laboratory overreads reclassified nearly 20% of the LVEF measurements.^61^ Accurately identifying and discriminating patients with HFmrEF with a narrow LVEF range (41%–49%) is particularly challenging in this context.^61^ The reliability of LVEF has also been questioned as it is related to loading conditions and hemodynamic statuses. Other imaging modalities such as global longitudinal strain look promising in better characterizing structural and functional abnormalities that are critical in the development of HF,^62^ but substantial variation (~5%–7%) between modalities still exists.^6^ Deep learning algorithms have shown potential in annotating 2D videos and Doppler modalities with similar accuracy and lower variability in automated compared to manual measurements.^63^ Automated measurements of LVEF could feature in future iterations of the universal definition as it appears to help improve diagnostic accuracy and overcome the variability issues ladened in manual measurements.

## 4 IMPLICATIONS FOR KEY STAKEHOLDERS

**Figure 3:**
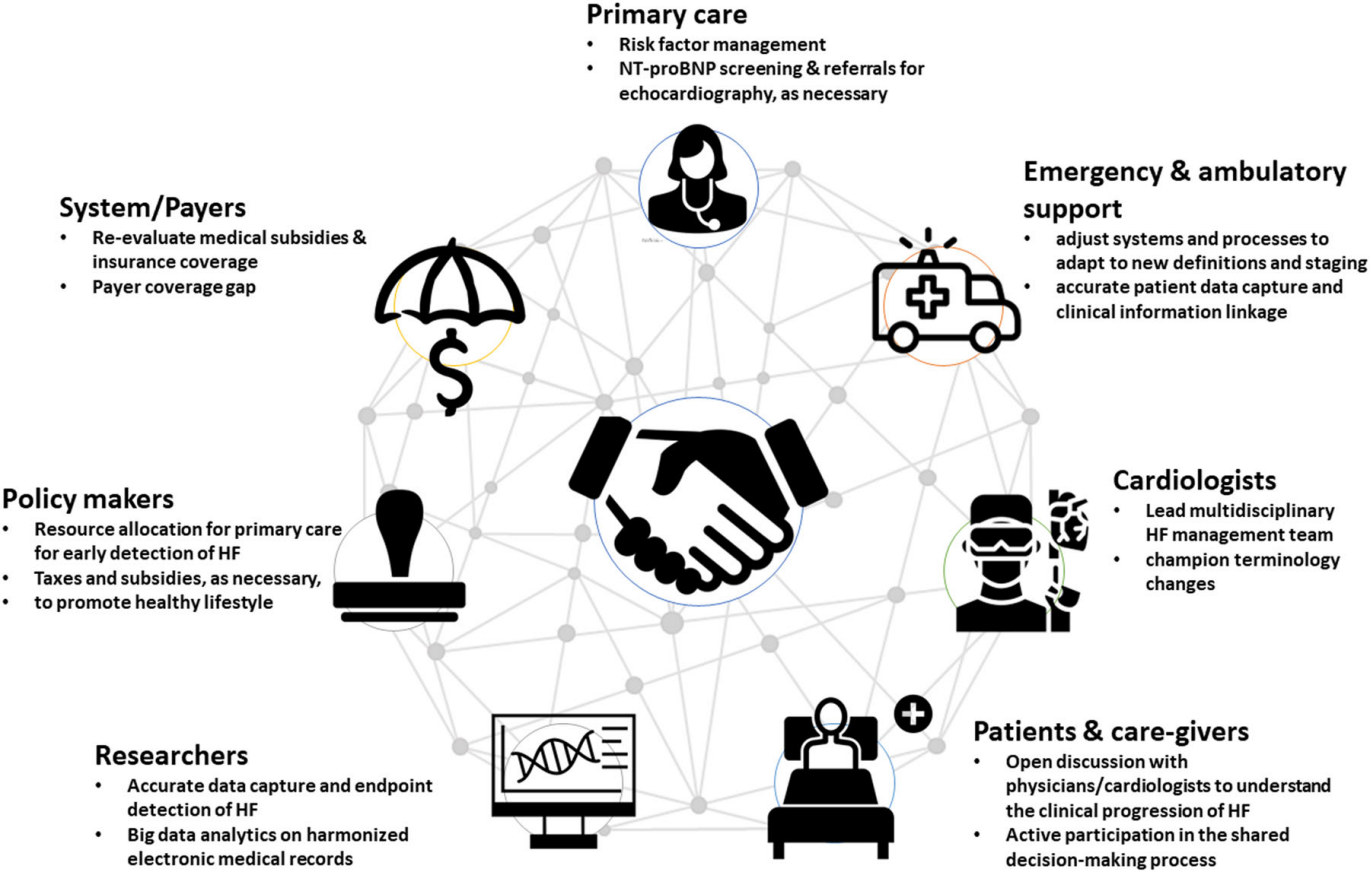
Clinical implications for key stakeholders.

This proposed universal definition provides a new platform for understanding, detection, assessment, treatment, and management of patients with HF. As demonstrated in Figure 3, this has important implications for broad range stakeholders—the most important of which are summarized.

### 4.1 Patients/caregivers

Any healthcare discussion with an individual at high risk of, or presenting with the syndrome should be focused on heart function, particularly in the early stages of the HF spectrum, that is, at‐risk and pre‐HF. The consensus document provides the opportunity for patient education and advocacy to clarify the stage at which an individual resides based on their actual clinical status. With a better understanding of the clinical progression of HF, patients/care providers may be more inquisitive about management approaches, facilitating shared decision‐making.^64^ Critically, the change in classification removes the stigma around “having HF” for many individuals, is less intimidating/frightening and potentially influences self‐care behavior as well as life insurance. Concurrently, a new emphasis away from “recovered HF” status is likely to promote greater adherence and less complacency among those living with the syndrome.

### 4.2 Primary care physicians

Individuals with undetected HF exist and continue to grow within our communities. This is largely attributable to a high level of misdiagnosis, missed diagnoses, disparities in access to healthcare, and late presentations to healthcare providers (especially relevant during the COVID‐19 pandemic).^65^, ^66^ Approximately one in six patients aged over 65 years presenting with breathlessness in primary care centres have unrecognized HF.^67^ Nearly 80% of all HF diagnoses are made following unscheduled hospitalizations despite 50% of patients having symptoms much earlier.^68^ The diagnosis of non‐acute HF in a primary care setting is notoriously difficult without echocardiography, especially in the early stages of HF. The universal definition recommends NT‐proBNP biomarker‐based screening to diagnose early stages of HF. Referrals for echocardiography may follow, as needed. Recent data from two large contemporary primary care studies of suspected HF showed that the NT‐proBNP cut‐off of 125 ng/L is diagnostically accurate in detecting new‐onset HF in a primary care setting.^69^ Primary care providers should be equipped to recognize patients in the early stages of HF (in the absence of symptoms) who need more proactive management to prevent and delay the onset of symptomatic HF in the future. How this is best achieved will need to be more fully explored from a research perspective and then articulated in expert clinical guidelines and reimbursement of coordinated activities of care.

### 4.3 Emergency and ambulatory care

Most patients presenting with acute HF are first evaluated and managed in emergency departments (EDs). Approximately one million annual ED visits in the United States are for acute HF, incurring nearly $39.2 billion in healthcare expenditure.^70^ Adjusting healthcare structures, systems, and processes in both ED setting and ambulatory care to promote the reach of the universal definition document will be required for effective implementation. Revision of medical record documentation will be necessary to capture the new definition, stages, and classification introduced. Accurate capture and linkage of clinical information during repeated visits will be key to understanding an individual's clinical trajectory.^64^

### 4.4 Cardiologists/HF specialists

Cardiologists and HF specialists are instrumental in championing the implementation of this universal definition. Although the ESC guidelines have emphasized the need for a multidisciplinary care to be provided for all patients, throughout the HF trajectory, from acute hospital admission to long‐term follow‐up, the level of adherence/adoption is still low, even in developed countries.^71^ Cardiologists should encourage the involvement of HF specialists' nurses, endocrinology, internal medicine, family medicine, emergency care specialists and pharmacists in developing a detailed HF management program, structured discharge planning, medication reconciliation, and follow‐up plans.^72^ Cardiologists can also champion the change toward the usage of more appropriate terminologies suggested in the consensus document. Initiating the use of terms will not only be technically more appropriate but also encourage more aggressive HF management. These include “new‐onset/de novo” HF for newly transitioning patients from pre‐HF to HF stage, “worsening HF” when HF signs and symptoms continue to deteriorate despite GDMT escalation, “persistent” instead of “stable” for lack of improvement, “remission” instead of “recovered” for previously symptomatic patients who have a resolution of symptoms and signs as well as resolution of previously present structural and functional heart disease.^6^

### 4.5 Researchers and allied health professionals

Standardized HF definitions allow clinical research data to be captured more accurately and extended beyond their current utility. Specific endpoint detection, such as HF hospitalization, will be feasible from electronic records if data are coded accurately. Harmonization of electronic medical records between regions will also be feasible and machine learning approaches can be utilized to identify cases of HF.^73^ The proposed universal definition separates HFmrEF from both HFrEF and HFpEF, recognizing that patients in this group may manifest heterogeneous features of either. Historically, patients now categorized as HFmrEF or improved have been understudied and often excluded from clinical trials testing new therapies for HF.^61^ Given the typical sex‐specific distribution of LVEF,^22^ this led to a bias toward male‐dominated trial cohorts. Further research is warranted to gather data on how the suggested terminology changes in the consensus document impact GDMT, shared decision making, patient education and treatment adherence and clinical outcomes.

### 4.6 Legislators and policymakers

Greater resource allocation for primary care and active annual screening for cardiovascular disease should be subsidized and supported. In the United States, the economic burden of the direct and indirect costs of HF in 2012 was US$30.7 billion, with nearly 80% of the cost associated with HF‐related hospitalization.^2^ Strategies for HF prevention and management are therefore extremely critical. The universal definition provides an excellent platform for early detection of individuals at risk and pre‐HF. Greater resource allocation to primary care will not only delay the onset of symptomatic HF but also drastically decrease the healthcare costs associated with managing HF patients in an advanced care setting.^74^ If resources allow, a relatively low actionable NT‐proBNP threshold will be desirable to identify a large number of people with mild to modest risk for primary prevention. For example, the Ministry of Health Singapore embarked on the “War on Diabetes” in 2016 to manage its growing disease burden with successful reward/incentive schemes that encourage healthier food choices and physical activity.^75^

### 4.7 System/payers

Despite strong evidence and clinical recommendations, the proportion of individuals receiving optimal GDMT is still low. Among 5000 chronic systolic HF patients enrolled in CHAMP‐HF (Change the Management of Patients With Heart Failure) registry, nearly two‐thirds discontinued GDMT or had no changes in dosage despite suboptimal therapy.^4^ One of the key reasons stated was the lack of medical subsidies and insurance coverage. Recalibration of healthcare systems and insurance providers, as well as adequate risk adjustment, will be required, especially for those in the early stages of HF (at risk and pre‐HF). Closing the payer coverage gap (ineligible for low‐income subsidies and 100% of drug cost is borne by patients) is also critical to ensure effective medication adherence and risk management.^76^ Proactive screening initiatives from health insurance companies to promote prevention and early detection of HF should be encouraged.

## 5 CONCLUSION

The concept of a universal definition of HF is a momentous leap forward in establishing an HF definition that is accepted worldwide, with the goal of early detection, and improving diagnosis and treatment globally. We applaud the Expert Committee of the Universal Definition and Classification of HF for this initiative. Specifically, the inclusion of raised natriuretic peptide levels in the diagnosis of HF elevates the diagnostic standards of HF to be more comprehensive, reliable, and objective. Recognition of patients' clinical trajectory changes in determining optimal therapies also holds critical clinical implications. The standardization of HF nomenclatures is not just a nicety, but a necessity, to avoid therapeutic complacency and promote aggressive management approaches. If implemented effectively, these changes will collectively influence the design of future clinical trials, interpretations of research data, clinician–patient conversations, patient care, and revamp HF management in the years to come.

## CONFLICTS OF INTEREST

Chanchal Chandramouli reports philanthropic research support from Lee Foundation Singapore and has received consultancy or speaker fees from Us2.ai, Boehringer Ingelheim, and Sanofi Aventis. Simon Stewart is supported by the NHMRC of Australia (GNT 1135894); has received speaking fees or honoraria from Edwards Lifesciences and Novartis; has received consultancy fees from Edwards Lifesciences (UK and Australia) and Novartis Australia; reports participation on a data safety monitoring board or advisory board at Edwards Lifesciences. Carolyn Su Ping Lam is supported by a Clinician Scientist Award from the National Medical Research Council of Singapore; has received research support from AstraZeneca, Bayer, Boston Scientific and Roche Diagnostics; has served as a consultant or on the Advisory Board/Steering Committee/Executive Committee for Actelion, Amgen, Applied Therapeutics, AstraZeneca, Bayer, Boehringer Ingelheim, Boston Scientific, Cytokinetics, Darma Inc., Us2.ai, Janssen Research & Development LLC, Medscape, Merck, Novartis, Novo Nordisk, Radcliffe Group Ltd., Roche Diagnostics, Sanofi, and WebMD Global LLC; and serves as cofounder and nonexecutive director of Us2.ai. Wael Almahmeed has nothing to declare.
